# Successful Photorefractive Keratectomy in a Case of Wilson's Disease

**DOI:** 10.1155/2021/6174130

**Published:** 2021-08-25

**Authors:** Saeed Shokoohi-Rad, Hamid-Reza Heidarzadeh

**Affiliations:** Eye Research Center, Mashhad University of Medical Sciences, Mashhad, Iran

## Abstract

**Purpose:**

To report a female with a history of Wilson's disease who underwent a successful photorefractive keratectomy (PRK) for myopic correction. *Case Presentation*. A twenty-year-old female with a history of Wilson's disease and D-penicillamine use was referred to our clinic for myopic refractive surgery. Her best-corrected visual acuity (BCVA) was 20/20 for both eyes with a refraction of ‐1.25‐0.5∗75° and ‐1.25‐0.25∗55° for the right and left eyes. The slit examination showed a prominent Kayser-Fleischer ring (K-F ring) in both eyes. She underwent a successful myopic PRK surgery, and her BCVA became 20/20 with no significant refraction.

**Conclusions:**

In this report, we report a successful PRK surgery for myopic correction in a case of Wilson's disease with prominent K-F rings in both eyes.

## 1. Introduction

Photorefractive keratectomy (PRK) is a Food and Drug Administration- (FDA-) approved refractive surgery for the correction of myopia, hyperopia, and astigmatism which employs a 193 nm argon fluoride excimer laser to ablate the anterior corneal stroma [1].

Wilson's disease is an autosomal recessive genetic disorder of copper metabolism caused by a mutation of the ATP7B gene, leading to copper accumulation in many tissues, mainly the liver, brain, cornea, and kidney [2]. The ocular manifestations include the Kayser-Fleischer ring (K-F ring) and sunflower cataract [3]. There has been no report that considers Wilson's disease a contraindication for refractive surgery, and this is the second case report of refractive surgery in a patient with Wilson's disease up to now.

Here, we describe a case of Wilson's disease with a prominent K-F ring who underwent a successful PRK for myopic correction.

## 2. Case Presentation

A twenty-year-old female with a history of Wilson's disease was referred to our clinic for refractive surgery. She was under treatment with D-penicillamine, an anticopper drug. Her best-corrected visual acuity (BCVA) was 20/20 for both eyes with a refraction of ‐1.25‐0.5∗75° in the right eye and ‐1.25‐0.25∗55° in the left eye. Keratometry was 43.25∗40°/43.5∗130° in the right eye and 43∗5°/43.5∗95° in the left eye. Her present glass and cycloplegic refractions were the same as BCVA. Slit-lamp examination showed prominent circumferential K-F ring in both eyes with clear lenses without any other pathologies. Intraocular pressure was 12 mm Hg without antiglaucoma medications in both eyes. Indirect ophthalmoscopy showed a 0.3 cup to disc ratio in both eyes without any other pathologies.

**Figure 1:**
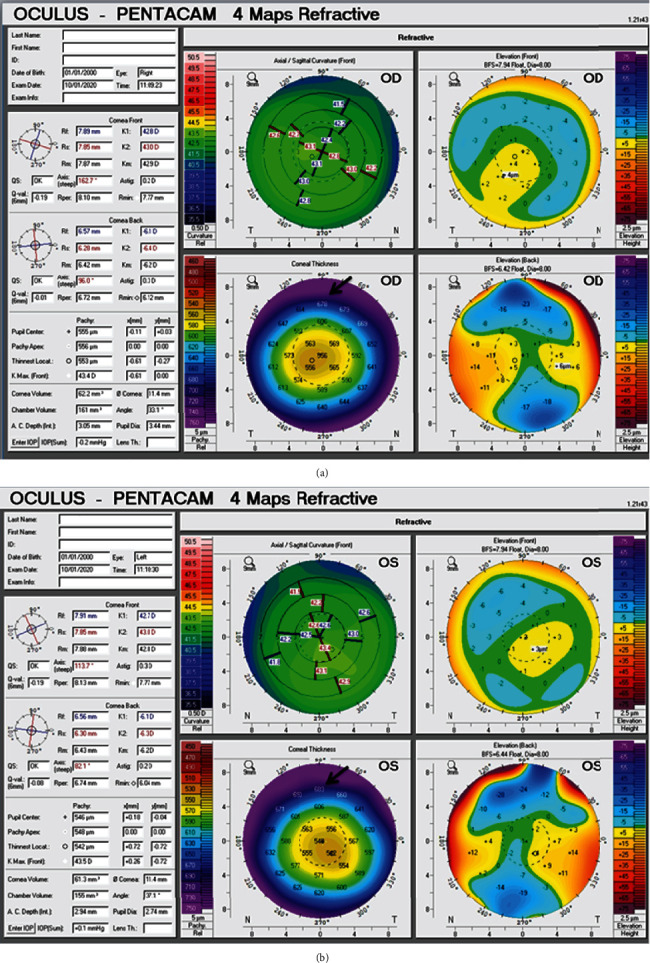
Corneal topography printouts of the right (a) and the left (b) eyes (Oculus-Pentacam). Corneal thickness maps show increased superior peripheral corneal thickness in both eyes due to copper deposition in Descemet's membrane (black arrows).

Corneal imaging studies performed and corneal topography (Orbscan II-Bausch & Lomb and Oculus-Pentacam) showed prolate corneal profile with a round pattern and enantiomorphism in both eyes. Pachymetry examination (Oculus-Pentacam) revealed the central corneal thickness to be 555 *μ*m in the right eye and 546 *μ*m in the left eye (Figure 1). There was no sign of keratoconus in patient corneal topography and tomography studies. In aberrometry imaging (Bausch & Lomb Zywave aberrometer), there was a central comma in both eye higher-order aberration (HOA), and predicted phoropter refraction (PPR) was ‐1.03‐0.69∗83° in the right eye and ‐1.21‐0.41∗83° in the left eye.

The PRK was performed using Technolas Perfect Vision GmbH (Bausch & Lomb). After topical anesthesia, under sterile conditions, a 7 mm diameter mark centered on the pupil was performed on the corneal surface. The epithelium was removed mechanically with a blunt scraper. The correction was preset at ‐2‐0.5∗75° for the right eye and ‐2.25–0.25∗55° for the left eye in the PROSCAN treatment mode. Maximum ablation was 52 *μ*m in the right eye and 55 *μ*m in the left eye within the treatment areas of 8.54∗8.2 mm in the right eye and 8.51∗8.67 mm in the left eye. After the procedure, levofloxacin solution was used and a therapeutic contact lens (TCL) was placed on both eyes. Betamethasone, levofloxacin single dose, and artificial tear drops were prescribed for the patient.

Seven days later, the patient visited for a follow-up. Corneal epithelium resurfacing was completed with no corneal haziness and TCL removed. Her uncorrected visual acuity was 20/20 in both eyes.

In 1 and 6 months after procedure follow-ups, her BCVA was 20/20 in both eyes with no significant refraction or residual refractive errors and her cornea showed no complication.

**Figure 2:**
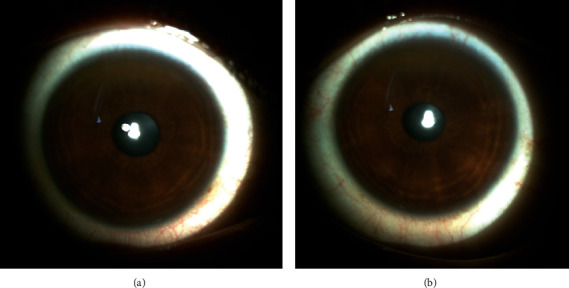
Slit-lamp photograph of the right eye (a) and the left eye (b) postoperation shows K-F ring in peripheral cornea, and the central area is clear.

**Figure 3:**
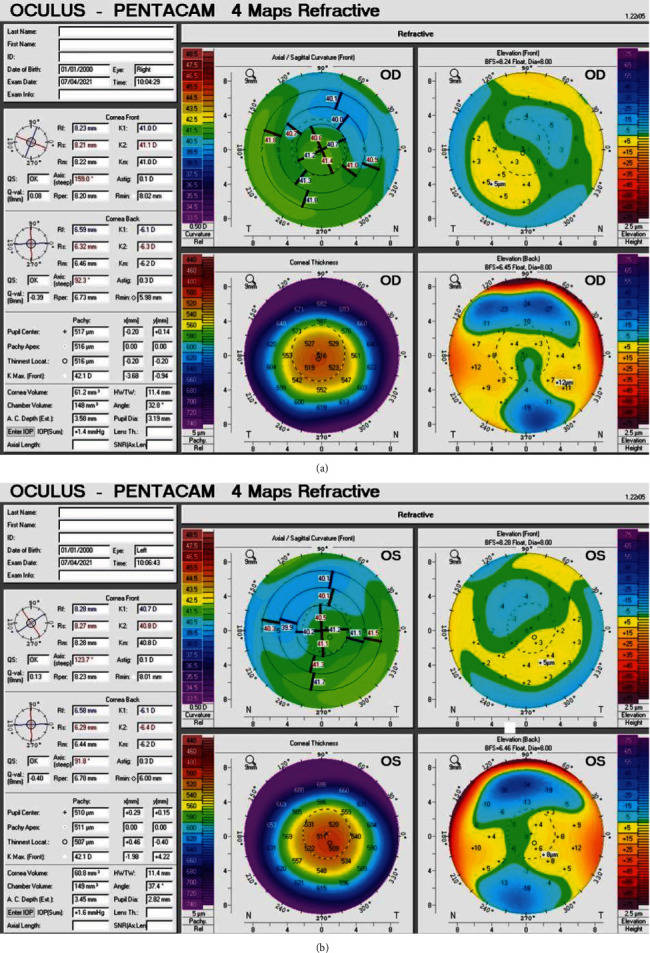
Postoperative corneal topography printouts of the right (a) and the left (b) eyes show no sign of corneal ectasia (Oculus-Pentacam).

Nine months later, her BCVA was 20/20 in both eyes with no significant refraction. The slit-lamp examination showed a prominent circumferential K-F ring in both eyes, and the cornea center was clear (Figure 2). In aberrometry imaging, there was a central comma in both eye HOA, and PPR was +0.37‐0.38∗92° in the right eye and +0.57‐0.19∗86° in the left eye. The Zernike root mean square was 0.13 *μ*m in the right eye and 0.15 *μ*m in the left eye, which shows that PRK did not induce HOA. In the Pentacam imaging, the central corneal thickness postoperation was 516 *μ*m in the right eye and 507 *μ*m in the left eye, and there was no sign of corneal ectasia in both eyes (Figure 3).

## 3. Discussion

PRK is a safe and effective refractive surgery option for correction of myopia up to -12 diopter (D), astigmatism up to 6 D, and hyperopia up to 5 D which use a 193 nm argon fluoride excimer laser to ablate the anterior corneal stroma [4].

Absolute contraindications of PRK are significant cataract, unstable glaucoma, corneal ectasias, thinning, edema, interstitial or neurotrophic keratitis, and extensive vascularization. Also, patients with active systemic connective tissue diseases such as systemic lupus erythematosus and rheumatoid arthritis have a contraindication for PRK due to the risk of corneal hazing and melting [1, 5].

PRK is relatively contraindicated in pregnant women, nursing mothers, functional monocularity, ocular conditions that limit visual function, excessively steep or flat corneas, abnormal corneal topography, significant irregular astigmatism, inadequately controlled dry eye, uveitis, glaucoma, history of herpes simplex keratitis, uncontrolled diabetes, and taking medications with a high risk of ocular side effects like isotretinoin and amiodarone [1, 5].

Typical ocular manifestations of Wilson's disease are K-F ring and sunflower cataract [3]. The K-F ring was described by the German ophthalmologists Bernhard Kayser and Bruno Fleischer [6]. At least 50% of patients and all patients with neurological disorder have this sign [7]. The extracellular copper depositions in Descemet's membrane of the cornea, which is called K-F ring, are almost bilateral and start primarily in the superior peripheral cornea, then inferior and later became circumferential [6]. Free copper loosely bound to albumin enters the aqueous humor, and the peripheral deposition in Descemet's membrane is attributed to the direction of the aqueous humor [8]. The K-F ring is reported to fade with anticopper therapy [9]. Theoretically, due to the depth and peripheral place of the K-F ring, it should not affect the laser beam, and the laser ablation zone is placed in the corneal central zone.

There is limited understanding of the safety of refractive surgery in patients with Wilson's disease. There has been just one report on PRK in a patient with Wilson's disease which was about undercorrection after PRK in a patient who had a prominent K-F ring like our patient, and it showed that PRK was performed safe and without any complication in that patient [10].

In conclusion, PRK showed to be a safe and effective refractive surgery for correction of myopia with no complication in our patient with a prominent K-F ring in both eyes due to Wilson's disease.

## Data Availability

The datasets used during the current study are available from the corresponding author on reasonable request.
